# Citrullinated myelin induces microglial TNFα and inhibits endogenous repair in the cuprizone model of demyelination

**DOI:** 10.1186/s12974-021-02360-3

**Published:** 2021-12-27

**Authors:** Miranda M. Standiford, Ethan M. Grund, Charles L. Howe

**Affiliations:** 1 Neuroscience PhD Program, Mayo Clinic Graduate School of Biomedical Sciences, Rochester, MN 55905 USA; 2 Translational Neuroimmunology Lab, Mayo Clinic, Rochester, MN 55905 USA; 3 Division of Experimental Neurology, Department of Neurology, Mayo Clinic, Rochester, MN 55905 USA; 4 Center for Multiple Sclerosis and Autoimmune Neurology, Mayo Clinic, Rochester, MN 55905 USA; 5 Present Address: Multiple Sclerosis and Neurorepair Research Unit, Biogen, Cambridge, MA 02142 USA

**Keywords:** Microglia, Myelin, Citrullination, TNFα, Remyelination

## Abstract

**Background:**

Microglia are the primary phagocytes of the central nervous system and are responsible for removing damaged myelin following demyelination. Previous investigations exploring the consequences of myelin phagocytosis on microglial activation overlooked the biochemical modifications present on myelin debris. Such modifications, including citrullination, are increased within the inflammatory environment of multiple sclerosis lesions.

**Methods:**

Mouse cortical myelin isolated by ultracentrifugation was citrullinated ex vivo by incubation with the calcium-dependent peptidyl arginine deiminase PAD2. Demyelination was induced by 6 weeks of cuprizone (0.3%) treatment and spontaneous repair was initiated by reversion to normal chow. Citrullinated or unmodified myelin was injected into the primary motor cortex above the cingulum bundle at the time of reversion to normal chow and the consequent impact on remyelination was assessed by measuring the surface area of myelin basic protein-positive fibers in the cortex 3 weeks later. Microglial responses to myelin were characterized by measuring cytokine release, assessing flow cytometric markers of microglial activation, and RNAseq profiling of transcriptional changes.

**Results:**

Citrullinated myelin induced a unique microglial response marked by increased tumor necrosis factor α (TNFα) production both in vitro and in vivo. This response was not induced by unmodified myelin. Injection of citrullinated myelin but not unmodified myelin into the cortex of cuprizone-demyelinated mice significantly inhibited spontaneous remyelination. Antibody-mediated neutralization of TNFα blocked this effect and restored remyelination to normal levels.

**Conclusions:**

These findings highlight the role of post-translation modifications such as citrullination in the determination of microglial activation in response to myelin during demyelination. The inhibition of endogenous repair induced by citrullinated myelin and the reversal of this effect by neutralization of TNFα may have implications for therapeutic approaches to patients with inflammatory demyelinating disorders.

**Supplementary Information:**

The online version contains supplementary material available at 10.1186/s12974-021-02360-3.

## Introduction

Multiple sclerosis (MS) is a devastating, immune-mediated disease of the central nervous system (CNS). During acute inflammatory attacks, MS presents with a diverse symptomology that results from reversible damage to the myelin sheath and transient disruption of saltatory conduction. While function may fully recover as inflammation resolves and endogenous myelin repair occurs, the temporary loss of myelin during such attacks renders axons vulnerable to injury via pathogenic immunological and metabolic processes. As the disease progresses, accumulative loss of axons eventually leads to irreversible disability. While current therapeutics are aimed at dampening or sequestering the immune response that damages myelin, active facilitation of remyelination is also crucial to protect vulnerable axons and prevent the accumulation of clinical deficits.

Microglia are key immune effectors in the CNS, producing a wide variety of cytokines, chemokines, and other soluble factors that exert both beneficial and detrimental effects on the repair capacity of the local microenvironment. During demyelination, damaged myelin must be cleared from the lesion in order for efficient remyelination to proceed [1–3]. Several studies have investigated microglial activation in response to myelin phagocytosis, but to date no consensus has been reached on the ultimate consequence(s) of myelin-laden microglia on the surrounding environment. While some studies have found an anti-inflammatory response to myelin [4–8], others have found a pro-inflammatory response [9–14] or no response at all [15]. Importantly, these studies did not specifically consider the biochemical composition of myelin as a determinant of the microglial response.

Biochemical myelin modifications that occur in MS alter the structure of myelin proteins, disrupt myelin compaction, and enhance the immunogenicity of myelin-derived epitopes. One such modification is citrullination, and MS patients exhibit increased levels of this modification throughout the brain [16–19]. Citrullination, also known as deimination, is the irreversible conversion of arginine to citrulline mediated by the calcium-dependent peptidyl arginine deiminases (PADs) [20]. Under homeostatic conditions, extracellular calcium levels are insufficient for PAD activation, but during inflammation and active myelin injury, calcium release from damaged cells drives PAD-mediated citrullination of arginine-rich proteins. Myelin basic protein (MBP) has 19 arginine residues in the 18.5 kDa isoform and conversion of these arginines to citrullines results in a loss of positive charge that inhibits myelin compaction and alters electrostatic interactions with other membrane-resident proteins and lipids [18, 21]. The cationicity and altered protein conformation induced by citrullination of MBP also confers greater susceptibility to degradation and enhances the generation of neoepitopes and altered immunodominant epitopes [22, 23].

Based on observations that there is increased citrullination of MBP in MS, particularly in fulminant MS lesions [19], we hypothesized that citrullination of proteins within myelin exerts a unique effect on microglia that results in exacerbation of demyelination or inhibition of repair. To test this hypothesis, we measured the influence of citrullinated (CIT) and unmodified (UNMOD) myelin on remyelination capacity in mice demyelinated by cuprizone and we investigated the phenotype induced in microglia by exposure to UNMOD or CIT myelin.

## Materials and methods

### Animal studies

All animal experiments were approved by the Mayo Clinic Institutional Animal Care and Use Committee and performed in accordance with institutional and National Research Council guidelines.

### Myelin isolation and modifications

Adult mice were euthanized and perfused with phosphate-buffered saline (PBS). Brains were weighed and homogenized in 20 volumes of 0.32 M sucrose. Samples were then underlaid with 12 mL of 0.85 M sucrose and ultracentrifuged for 45 min at 95,000 g. Myelin was isolated from the middle layer of the gradient. For citrullination, myelin was incubated for 3 h at 37 °C with 20 µg PAD2 (Sigma, #SRP5224) in buffer containing 20 mM Tris–HCl (pH 7.3), 0.3 M NaCl, 1 mM ethylenediaminetetraacetic acid (EDTA), 10 mM dithiothreitol (DTT), and 5 mM CaCl_2_. The reaction was terminated by quenching with 0.5 M EDTA. The crude myelin mixture was resuspended in cold water and ultracentrifuged at 95,000 g for 45 min. The pellets were resuspended in 1 mL Ca^2+^ and Mg^2+^ free PBS. Total protein was quantified using Pierce protein assay kit (Thermo Scientific, #23227).

### Mixed glia culture

Postnatal pups (age P1–P3) were used for primary cultures. Briefly, cerebral hemispheres were collected and meninges removed. Tissue was minced and digested with a final concentration of 0.025% trypsin at 37 °C for 30 min on a shaker. The reaction was stopped by addition of 10% fetal bovine serum (FBS), 1 mg/mL DNase I, and 3.82% MgSO_4_. Tissue was then placed on ice for 10 min followed by centrifugation at 1000 rpm for 5 min. Being careful to not introduce bubbles, cell pellets were resuspended in Earle’s Balanced Salt Solution containing hydroxyethyl piperazineethanesulfonic acid, MgSO_4_, DNase I, and FBS and underlaid with 4% bovine serum albumin (BSA). Cells were centrifuged at 100 g for 8 min without braking. Cells were resuspended in Dulbecco’s Modified Eagle Medium (DMEM) (Corning, #10-013-CV) with 10% FBS and 1X penicillin–streptomycin–glutamine (Gibco, #10378-016) and plated at 10^7^ cells/T75 flask previously coated with poly-L-lysine. Media was replaced every other day until microglia isolation.

### Microglia isolation and treatment

Two weeks after preparation of mixed glia cultures, microglia were isolated using an immunomagnetic positive selection kit targeting CD11b (StemCell Technologies, #18970) following manufacturer’s instructions. Briefly, cells were trypsinized and triturated to break up cell aggregates before passing cells through a 70 µm cell strainer and centrifuging at 200 g for 5 min. Cells were then resuspended at 10^8^ cells/mL in PBS containing 2% FBS and 1 mM EDTA and incubated with 50 µL/mL of the selection cocktail (25 µL component A and 25 µL component B) for 5 min at room temperature. Rapidspheres were added at 80 µL/mL and incubated for 3 min. The total volume was brought up to 2.5 mL and placed on a magnet for 10 min. The negative fraction was discarded and the remaining cells were again resuspended in 2.5 mL media and placed on the magnet for 5 min. This was repeated twice. After isolation of the pure microglia population, cells were resuspended in DMEM:Nutrient Mixture F12 (Gibco, #11320-033) containing 10% FBS, 1 × penicillin–streptomycin (Gibco, #15140-122), 2 mM L-Glutamine (Gibco, #25030-081), 5 µg/mL N-acetyl-L-Cysteine (Sigma, #A9165), 5 µg/mL insulin (Sigma, #I6634), 100 µg/mL apo-transferrin (Sigma, #T1147), 100 ng/mL sodium selenite (Sigma, #S5261) and 20% LADMAC conditioned media (a source of colony stimulating factor 1). Microglia were cultured for 5 days prior to treatment. Microglia were treated with 50 μg/mL myelin or an equivalent volume of PBS as vehicle.

### OPC proliferation assay

Primary microglia were treated with unmodified myelin or citrullinated myelin for 24 h. Following a complete media change to remove myelin debris, iPSC-derived OPCs (courtesy of the laboratory of James Dutton) [24] were plated on top of myelin-fed microglia. Twenty-four hour post-co-incubation, OPC proliferation was assessed using the Click-IT Plus EdU cell proliferation kit (ThermoFisher, C10638) according to the manufacturer’s protocol.

### Cuprizone diet

We used 0.3% cuprizone chow manufactured by TestDiet using bis(cyclohexanone)oxaldihydrazone (Sigma, #14690). Animals were administered cuprizone chow for 6 weeks ad libitum. Animals were then removed from cuprizone chow and injected intracerebrally with myelin or vehicle and returned to standard chow for 3 weeks to allow for remyelination.

### Intracerebral myelin injection

Anesthetized adult mice (> 8 weeks of age) were intracerebrally injected with 50 µg of myelin (5 µL total volume of 10 µg/µL myelin sample) at a rate of 0.5 µL/min using a 10 µL, 26-gauge, 2 inch, point style 3, model 1701 N Hamilton syringe. The stereotactic coordinates for the injection site were + 1.1 mm,  + 1.5 mm from bregma at a depth of 1.5 mm in the primary motor cortex above the corpus callosum.

### CX3CR1^CreER^ x Rpl22 (Ribotag) RNA isolation and RTPCR

B6.129P2(C)-Cx3Cr1tm2.1(cre/ERT2)Jung/J (Jackson Laboratory, #020940) mice were crossed with B6N.129(Cg)-Rpl22tm1.1Psam/J (Jackson Laboratory, #011029) mice and will be referred to as Cx3Cr1CreER x Rpl22 mice. Tamoxifen (Sigma, #10540-29-1) was prepared at a concentration of 20 mg/mL in corn oil. Four-week-old Cx3Cr1^CreER^ x Rpl22 ribotag mice were injected intraperitoneally with 100 µL tamoxifen for five consecutive days. Six weeks following recombination mice were injected intracerebrally and tissue isolated 24 h post injection. Mice were euthanized and perfused with PBS containing 0.1 mg/mL cycloheximide. A 4 mm biopsy punch was used to isolate the injection site, only taking the cortical layer. This tissue was then homogenized in 10% w/v supplemented homogenization buffer (49.5 mM Tris, 100 mM KCl, 12 mM MgCl2, 1% NP-40 supplemented with 1 mg/mL heparin, 0.1 mg/mL cycloheximide, 1 mM DTT, RNAsin, and protease inhibitors). Samples were spun at 10,000 g for 10 min and a maximum of 800 µL was used for RNA isolation. Each sample was then incubated with 3 µL anti-hemagglutinin.11 antibody (2 mg/mL; Biolegend, #901503) for 4 h with gentle agitation at 4 °C. Samples were incubated overnight with 200 µL of Dynabeads Protein G (Invitrogen, #10004D). Samples were placed on a magnet and the negative fraction removed. The magnetic beads were washed three times with a high salt buffer (49.5 mM Tris, 300 mM KCl, 12 mM MgCl2, 1% NP-40, with 1 mg/mL heparin, 0.1 mg/mL cycloheximide, 1 mM DTT). RNA was then removed from beads using 350 µL Buffer RLT containing 2-mercaptoethanol and isolated using the Qiagen RNeasy Micro Kit (Qiagen, #74004). RTPCR was analyzed using the BioRad PrimePCR PreAmp system according to the manufacturer’s protocol. Primers: CCL5 (Unique Assay ID: qMmuCID0021047), TNFα (Unique AssayID: qMmuCID0004141), Glyceraldehyde-3-phosphate dehydrogenase (Gapdh) (Unique Assay ID: qMmuCID0027497).

### TNFα neutralization

Mice received 0.5 mg of anti-TNFα IgG (Bio X Cell, #BE0058) or isotype control (Bio X Cell, #BE0088) by intraperitoneal injection on days 0, 1, 2, and 3 post-myelin injection and 0.2 mg of anti-TNFα IgG or control on days 5, 7, 9, 11, 13, and 15 post-injection. Animals were euthanized 21 days after myelin injection for analysis. The TNFα neutralization scheme was modeled after published experiments [25–27] and administration was extended over the 2 week period to ensure breakthrough did not confound the final analysis.

### Cytometric bead array

TNFα levels were measured in microglial supernatants and tissue lysates using a cytometric bead array (CBA) assay. CBA flex sets (TNFα, #558299; CCL5, #558345) and reagent kits (#560006) from BD Biosciences were used according to manufacturer’s instructions. The enhanced sensitivity TNFα CBA (#562336) was used for cortical lysates. For this analysis, injection sites were isolated using a 4 mm diameter biopsy punch and only the cortical layers were analyzed. Samples were homogenized in PBS containing protease inhibitors and clarified prior to analysis.

### Flow cytometry

Primary microglia were blocked for 30 min in PBS containing Ca^2+^ and Mg^2+^ with 1% BSA and 2.4G2 antibody. Cells were labeled with antibodies (CD11b #56114, MHCII ab25584-100, CD80 #553769, CD86 #558703) for 1 h, then washed and resuspended in block buffer and analyzed using a BD Accuri C6 Plus cytometer.

### pHrodo labeling and myelin quantification in vitro

Myelin was labeled with the pH sensitive dye pHrodo red succinimidyl ester (Invitrogen, #P36600), as previously described [28]. pHrodo-labeled myelin was used to estimate myelin uptake. Microscopic images of pHrodo signal were converted to a binary image using ImageJ and total positive pixels were counted as a measurement of myelin uptake.

### RNAseq

RNA was isolated from primary microglia using the Qiagen RNeasy Mini Kit (Qiagen, #74104). 200 ng of total RNA was submitted for analysis. mRNA library preparation was done using TruSeq RNA Library Prep Kit v2 (Illumina, #RS-122-2001). Sequencing was performed using next generation sequencing on the Illumina HiSeq4000 platform, with paired end, 100M reads. The raw RNA sequencing of paired-end 101 × 2 reads was processed through the Mayo RNA-Seq bioinformatics pipeline, MAP-RSeq version 3.0.1 [29]. MAP-RSeq employs the very fast, accurate and splice-aware aligner, STAR [30] to align reads to the reference mouse genome build mm10. Using aligned reads, gene and exon expression quantification were performed using the Subread [31] package to obtain normalized (reads per kilobase per million mapped reads—RPKM) values. Gene sets were reduced to targets with at least one expression value > 1.0 in any condition. Following two-way ANOVA each gene and each condition were analyzed by individual *t* test using the two-stage linear step-up procedure of Benjamini, Krieger and Yekutieli [32]. Genes from each comparison were ranked by false discovery rate (FDR) *q* value and only genes with FDR < 5% were retained for the comparisons between unmodified myelin and vehicle and between citrullinated myelin and vehicle; comparison between unmodified and citrullinated myelin used FDR < 20%. The retained genes were again ranked by FDR *q* value (-log10) and used as input to the GSEA 4.1.0 algorithm [33, 34]. Hallmark enriched pathways were identified for each condition, ranked by FDR *q* value, and retained if FDR < 20%. From the retained pathways, raw RPKM values from 691 genes were loaded into Heatmapper [35] and clustered by average linkage and Pearson distance with color assigned by z-score. Genes that clustered based on elevated z-score in the citrullinated group relative to both vehicle and unmodified myelin groups were selected and analyzed by one-way ANOVA. Following Tukey’s pairwise comparison 25 genes were retained.

### Western blot

Samples were first lysed in 1% NP-40, 0.5% deoxycholic acid sodium salt, and 10% glycerol lysis buffer (20 mM Tris, pH 8) and then mixed 1:1 with 2X Laemmli sample buffer with 2-mercaptoethanol. Samples were boiled for 5 min prior to loading into Criterion Tris–HCl gels (Bio-Rad). Samples were run and transferred using the Bio-Rad Criterion system. Primary antibodies were used as directed. Secondary antibodies were used at 1:5000. Blots were developed using the SuperSignal West Pico PLUS Chemiluminescent substrate (Thermo Scientific, #34577) and imaged on a Bio-Rad ChemiDoc. Antibodies: Antibodies: anti-peptidyl-citrulline (1:250; Millipore, #MABN328), anti-PAD2 (1:1000; Caymen Chemical, #19822), anti-MBP (1:500; Sigma, #MAB386).

### Immunofluorescence

All cells and tissues were fixed with 4% paraformaldehyde. Tissues and cells were incubated for 1 h in blocking buffer (5% serum of the secondary host species, 1% BSA, 0.1% Triton X100 in PBS with Ca^2+^ and Mg^2+^). Samples were incubated overnight with primary antibody (anti-MBP Millipore #MAB386 at 1:500; anti-Iba-1 Wako #019-19741 at 1:200; anti-TNFα Abcam AB9739 at 1:100) at 4 °C. The following day, samples were thoroughly washed with PBS with Ca^2+^ and Mg^2+^ before incubating with fluorescently labeled secondary antibody (1:500) in the dark at 37 °C for 1 h. Samples were washed thoroughly with PBS with Ca^2+^ and Mg^2+^ then coverslipped with Vectashield antifade mounting medium containing DAPI (Vector Labs, H-1200). MBP surface area was quantified using Neurolucida 360 software (MBF Bioscience). For Tmem119 (1:500, Cell Signaling #90840S) colocalization with Iba-1 (1:500, Wako #015-28011), samples were blocked for 1 h before staining overnight with Tmem119 primary antibody following the above protocol. Samples were subsequently washed and labeled with an Alexa488-conjugated anti-rabbit secondary antibody, followed by thorough washing and another incubation in blocking buffer at room temperature for 1 h. Sections were then incubated overnight at 4 °C with a rabbit anti-Iba-1 antibody directly conjugated to SPICA568. Sections were washed and mounted as above.

### Statistical analysis

Statistical analyses were performed using Prism 9 (GraphPad). Normality was determined using the Shapiro–Wilk test and normally distributed data were checked for equal variance. Parametric tests were applied to data that were normally distributed and of equal variance. Tukey’s multiple comparison test was used for post hoc comparisons following ANOVA.

## Results

### Intracerebral injection of citrullinated myelin reduces spontaneous remyelination in the cuprizone model

**Fig. 1:**
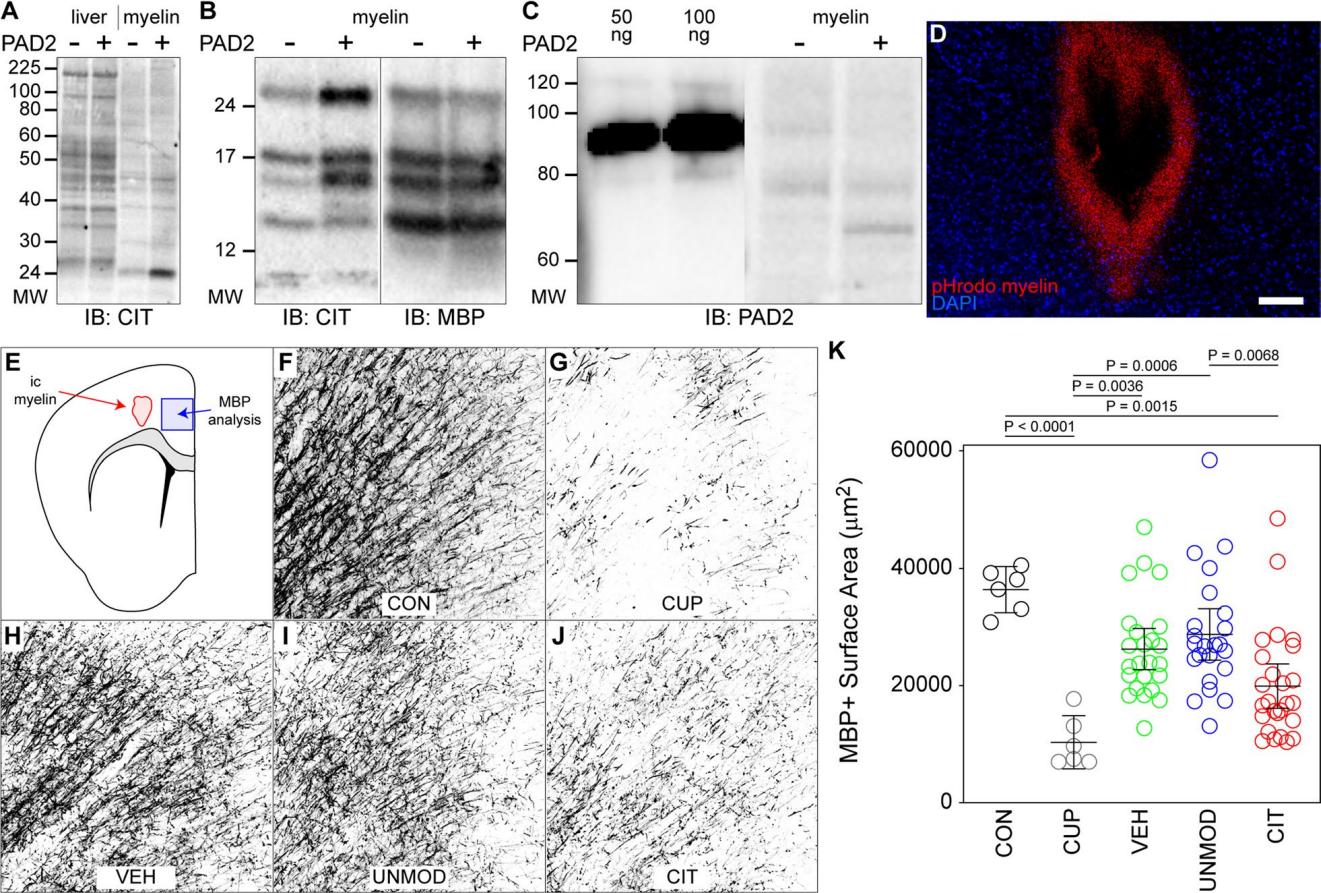
Citrullinated myelin inhibits remyelination. **A** Western blot analysis of liver and myelin preparations citrullinated ex vivo by incubation with PAD2 in the presence of high calcium. **B** Western blot analysis of unmodified and citrullinated myelin probed for citrulline (CIT) and myelin basic protein (MBP). **C** Analysis of PAD2 enzyme carry-through in the modified myelin preparation. **D** Evidence that pHrodo red-labeled myelin is phagocytosed by microglia around the injection site following stereotactic delivery of 10 μg myelin above the corpus callosum. Scale bar is 100 μm. **E** Schematic showing site of intracranial myelin injection and region of analysis for MBP surface area. Representative images of MBP-labeled tracts radiating from the cingulum bundle in **F** control (CON) mice that were never demyelinated, **G** cuprizone (CUP)-fed mice at the end of the demyelination phase, **H** mice that received vehicle (VEH) injection, **I** mice that received unmodified (UNMOD) myelin, and **J** mice that received citrullinated (CIT) myelin by intracranial injection. **K** Quantitative Neurolucida analysis of MBP surface area along fibers within a 0.4 mm^2^ ROI. Each symbol represents one animal; CON, *n* = 6; CUP, *n* = 6; VEH, *n* = 24; UNMOD, *n* = 23; CIT, *n* = 26. Graph shows mean ± 95% CI. Pairwise comparisons not shown are insignificant

We isolated myelin from the brain of adult mice using gradient centrifugation and induced citrullination ex vivo using recombinant PAD2 in the presence of high levels of calcium. Relative to liver tissue, PAD2 treatment of myelin led to increased citrullination of specific proteins (Fig. 1A), indicating that the ex vivo biochemical modification was not indiscriminate and suggesting that only certain proteins are susceptible to detectable citrullination. Within myelin, the strongest citrullination signal was detected on several low molecular weight bands. Of these, two predominant bands comigrated with MBP isoforms (Fig. 1B). Quenching and dilution of the myelin preparation following citrullination was sufficient to remove residual PAD2 enzyme that could potentially confound downstream analyses (Fig. 1C). Myelin was labeled with pHrodo red, a pH-sensitive molecule that is weakly fluorescent at neutral pH and strongly fluorescent at the acidic pH found within phagosomes. Injection of this labeled myelin into the primary motor cortex resulted in robust phagocytosis by cells around the injection site (Fig. 1D), indicating that the preparation was a suitable source of myelin debris that could be introduced into the brain as a surrogate for damaged myelin.

Our primary question was whether citrullinated myelin impacted the endogenous repair processes engaged following demyelination. Mice were, therefore, fed cuprizone-containing chow (0.3%) for 6 weeks to induce stable demyelination of the corpus callosum and associated myelin tracts (Fig. 1G). Mice were then switched to normal chow for 3 weeks to initiate spontaneous remyelination. Within this paradigm, vehicle (VEH, PBS), UNMOD myelin, or CIT myelin was stereotactically injected (50 μg in 5 μL) into the primary motor cortex just above the cingulum bundle (Fig. 1E) at the time animals were switched to normal chow. Three weeks later the extent of remyelination along the axons radiating from the cingulum was assessed by immunostaining for MBP (Fig. 1F–J). We assessed remyelination in this region because the cortex has similar demyelination and remyelination dynamics as the corpus callosum during cuprizone administration but is even more profoundly demyelinated [36]. The axons in this region project through the site of our myelin injection and distinct fibers are visible, allowing finer resolution of repair than is possible in the tightly bundled corpus callosum. Finally, by assessing remyelination at 3 week post-injection, any myelin debris we injected was cleared, eliminating potential confounding effects on our analysis of myelin intensity. The total area of MBP positivity within a 0.4 mm^2^ region was measured, revealing that injection of UNMOD myelin had no effect on remyelination compared to VEH-injected mice (*F*(4,80) = 9.921, *P* < 0.0001 by one-way ANOVA across all conditions; VEH vs UNMOD: *P* = 0.8681 by Tukey’s pairwise comparison) (Fig. 1K). In contrast, remyelination in mice receiving CIT myelin was substantially lower relative to UNMOD and VEH (CIT vs UNMOD: *P* = 0.0068 by Tukey’s pairwise comparison). Notably, while VEH (*P* = 0.1970) and UNMOD (P = 0.3317) animals exhibited MBP levels that were statistically similar to mice that were never demyelinated (CON; Fig. 1F), CIT mice remained significantly demyelinated 3 weeks after injection (P = 0.0009). In fact, the amount of MBP measured in CIT mice was not different from the level measured in animals that were examined at the end of the 6 week cuprizone chow (not switched to control chow; CIT vs CUP: *P* = 0.1231). This effect was not due to an impact on oligodendrocyte precursor cell (OPC) proliferation, as CIT myelin-laden microglia did not change OPC proliferation in vitro relative to UNMOD myelin (Additional file 1: Fig. S1). We conclude that citrullinated myelin impairs the capacity for the cortex to spontaneously remyelinate following cessation of cuprizone toxicity.

### Intracerebral injection of myelin induces microglial activation

**Fig. 2:**
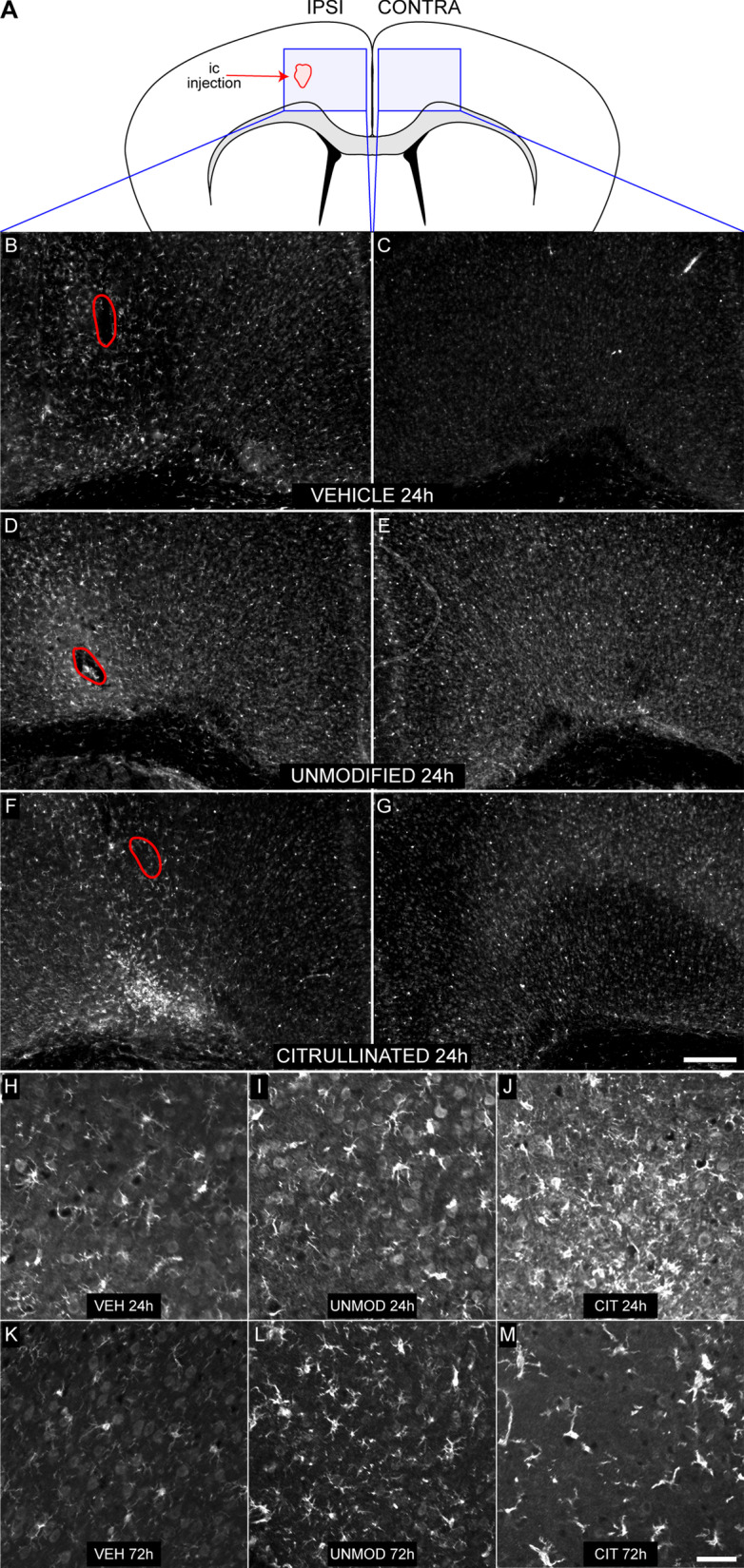
Intracranial injection of myelin activates microglia. **A** Schematic showing the site of intracranial injection and the region of analysis for microglial activation. **B**–**M** Tissue sections collected at 24 or 72 h post-injection were immunostained against Iba-1. Representative low magnification images from ipsilateral **B**, **D**, **F** and contralateral **C**, **E**, **G** cortex are shown from animals injected with vehicle (VEH) (**B**, **C**), unmodified myelin (UNMOD) (**D**, **E**), or citrullinated myelin (CIT) (**F**, **G**). Representative higher magnification images at 24 h **H**–**J** and 72 h **K**–**M** postinjection for mice receiving VEH (**H**, **K**), UNMOD myelin (**I**, **L**), or CIT myelin (**J**, **M**). Scale bar in (**G**) is 250 μm and refers to (**B**–**G**); scale bar in **M** is 50 μm and refers to (**H**–**M**)

Given the role for microglia in phagocytosis of myelin debris, we hypothesized that the differential effect of UNMOD and CIT myelin on remyelination was associated with more microglial activation in response to the modified material. Employing the same strategy as above, myelin was injected above the cingulum bundle in healthy mice (Fig. 2A). Tissue sections collected 24 or 72 h after injection were immunostained with antibody against Iba-1 to reveal activated microglia. Mice receiving vehicle injections (PBS) showed mild reactivity in the ipsilateral cortex (Fig. 2B, H, K) and no response in the contralateral cortex (Fig. 2C). Unexpectedly, mice receiving either UNMOD myelin or CIT myelin showed strong microglial reactivity in the ipsilateral cortex (Fig. 2D, F) and increased reactivity in the contralateral cortex (Fig. 2E, G). While reactivity at 24 h may have been higher in the CIT injected animals (Fig. 2J) relative to the UNMOD recipients (Fig. 2I), by 72 h the level of reactivity was similar between the groups (Fig. 2L, M). While it is possible that citrullinated myelin exerted an inhibitory effect on remyelination due to more overall microglial reactivity, the degree of such reactivity in mice receiving unmodified myelin and the overall variability in the degree and extent of reactivity in either group argues against such a model. Finally, given that Iba-1 reactivity is not specific to microglia and to rule out a substantial role for infiltrating monocyte-derived cells, we colocalized Iba-1 with Tmem119 in animals injected with CIT myelin. By 72 h after injection the majority of Iba-1 positive cells clearly exhibited Tmem119 immunoreactivity indicative of microglial phenotype (Additional file 1: Fig. S1). We conclude that both unmodified and citrullinated myelin induce microglial reactivity, and therefore an absence of microglial response to unmodified myelin cannot explain the differential effect of citrullination on endogenous repair mechanisms.

### Microglial phagocytosis and degradation of myelin are not impacted by citrullination

**Fig. 3:**
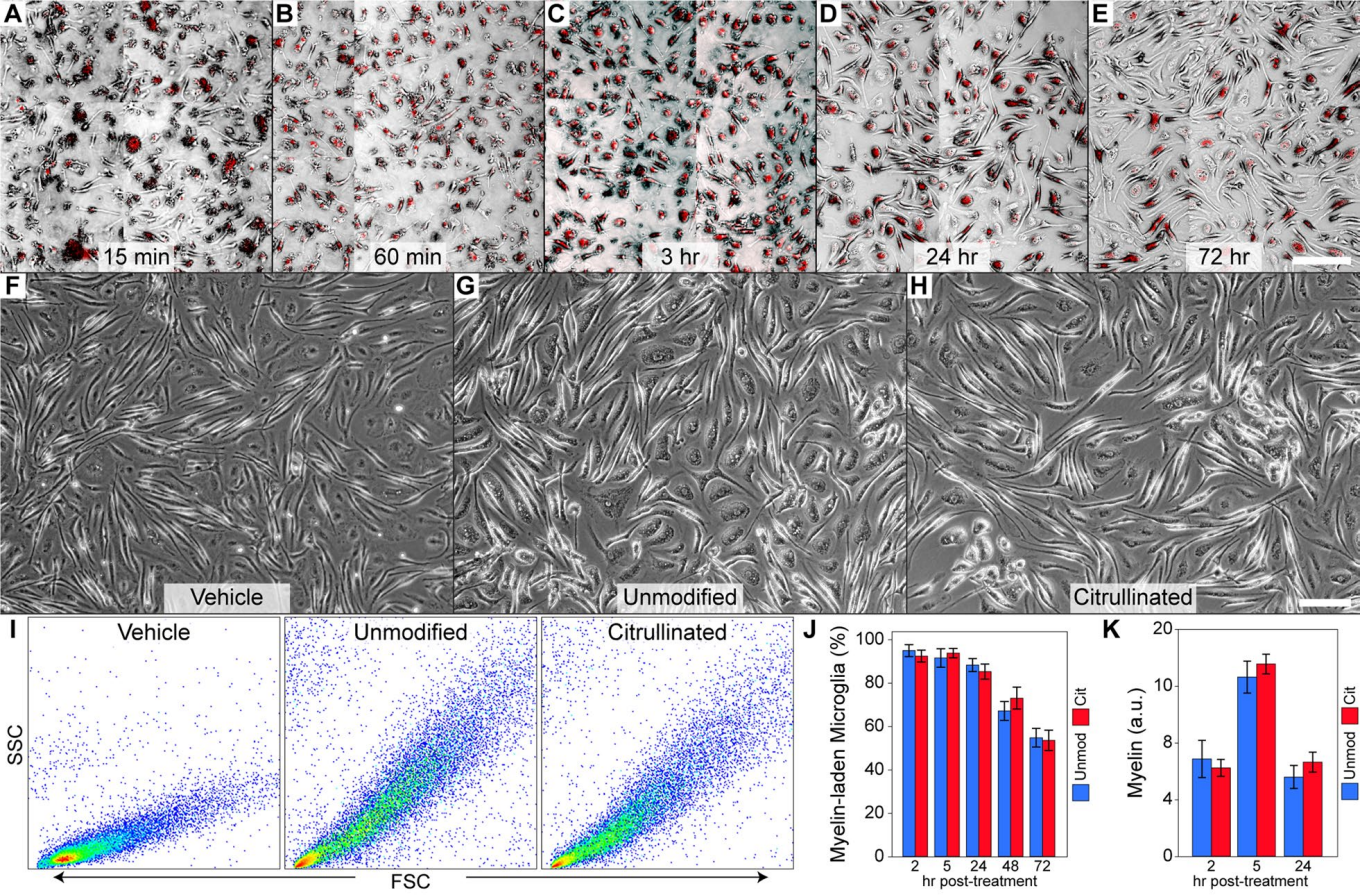
Microglial phagocytosis of myelin is not impacted by citrullination. **A** pHrodo red-labeled myelin is phagocytosed within 15 min of addition to primary microglia (red = phagocytosed myelin; cells shown in brightfield). **B**–**C** Phagocytosis continues through 3 h. **D** By 24 h the total amount of myelin decreased and the cells acquired a larger and more reactive morphology. **E** Morphological change is maintained through 72 h. **F** Vehicle-treated cells remain small and largely bipolar. **G**, **H** Incubation for 24 h with either unmodified (UNMOD) or citrullinated (CIT) myelin results in the same morphological change relative to vehicle. **I** Flow cytometric analysis of forward and side scatter profile indicates that both UNMOD and CIT myelin trigger a robust change in cellular size and morphology. **J** Percent of myelin-laden microglia is the same between UNMOD and CIT conditions. **K** Uptake and degradation rates are the same for UNMOD and CIT myelin. Graphs show mean ± 95% CI. Scale bar in **E** is 200 μm and refers to (**A**–**E**). Scale bar in **H** is 100 μm and refers to (**F–H**)

To further characterize and monitor the microglial response to CIT myelin we isolated primary microglia under conditions that promoted a relatively unreactive state in culture. These cells were incubated with pHrodo red-labeled myelin (50 μg/mL total protein) and then live-imaged at different timepoints to assess uptake (Fig. 3A–E, J). Red signal, indicative of internalization, was evident as early as 15 min after incubation (Fig. 3A); by 3 h most microglia contained phagocytosed myelin (Fig. 3C). By 24 h the number of cells showing red signal decreased and the morphology of the cells was consistent with acquisition of a reactive phenotype (Fig. 3D); this pattern continued through 72 h (Fig. 3E). Relative to vehicle-treated (PBS) microglia (Fig. 3F), cells treated with either UNMOD (Fig. 3G) or CIT myelin (Fig. 3H) exhibited morphological changes consistent with reactivity. Flow cytometric analysis confirmed the robust phagocytosis and morphological change associated with either UNMOD or CIT myelin at 24 h (Fig. 3I). Quantitation of the percent of microglia exhibiting signal for phagocytosed myelin at different timepoints indicated no difference in the rate of uptake between UNMOD or CIT material (Fig. 3J). Likewise, the total amount of phagocytosed material did not differ between the preps, indicating that the rate of degradation was unchanged by the modification (Fig. 3K). We conclude that microglial uptake and degradation of myelin and acquisition of a reactive morphology in response to myelin are not impacted by citrullination, indicating that the differential repair response observed in vivo was not the result of different upstream microglial responses.

### Citrullinated myelin induces a unique microglial transcriptional signature

**Fig. 4:**
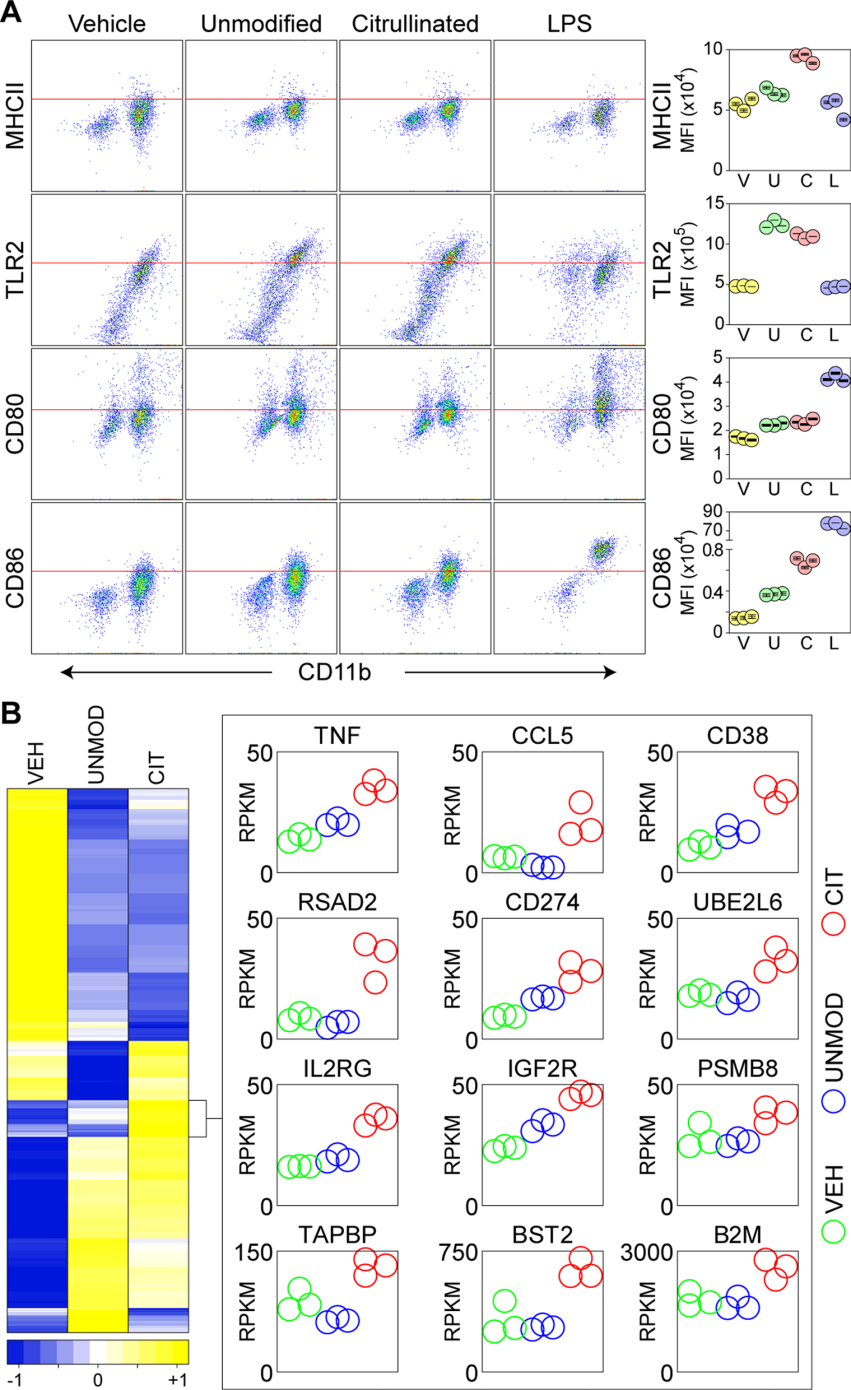
Citrullinated myelin induces a unique transcriptional program in microglia. **A** Primary microglia were stimulated with vehicle (VEH), unmodified myelin (UNMOD), citrullinated myelin (CIT), or LPS for 24 h and then analyzed by flow cytometry for surface level expression of the activation markers MHCII, TLR2, CD80, and CD86. Red lines provide reference for expression intensity across groups. Graphs show individual experiments (*n* = 3 for each condition) with mean and standard deviation: V = vehicle; U = unmodified; C = citrullinated; L = LPS. **B** Heatmap showing z-scores for 691 unique genes clustered by average linkage and Pearson distance. Graphs show reads per kilobase per million mapped reads (RPKM) for a subset of 12 genes with elevated z-scores in microglia treated with CIT myelin relative to both VEH and UNMOD myelin

Despite the similarity in response profile induced by UNMOD and CIT myelin, we hypothesized that citrullinated myelin must induce some sort of unique response in microglia. We assessed changes in several activation-related surface markers in primary microglia exposed to vehicle (PBS), UNMOD myelin (50 μg/mL), CIT myelin (50 μg/mL), or lipopolysaccharide (LPS) (100 ng/mL) for 24 h (Fig. 4A). While CIT myelin induced upregulation of MHCII and CD86 above the level induced by UNMOD myelin, the effect size of these responses was modest, with less than a doubling in either surface marker. Since these surface markers represent a reactive state but do not convey information about effector function, per se, we characterized the transcriptional response induced by myelin using RNAseq. Two-way ANOVA of the normalized transcript counts acquired from microglia incubated for 24 h with vehicle (*n* = 3), UNMOD myelin (*n* = 3), or CIT myelin (*n* = 3) revealed a highly significant difference between groups (*F*(27,368, 82,110) = 1.775, *P* < 0.0001). Using the two-stage linear step-up procedure of Benjamini, Krieger and Yekutieli [32] to provide false discovery rate correction for the large number of comparisons, we found 8094 genes upregulated in response to UNMOD myelin relative to vehicle (FDR < 5%) and 8558 genes upregulated in response to CIT myelin relative to vehicle (FDR < 5%). Comparison of the CIT myelin response to UNMOD myelin indicating 1826 genes that were differentially impacted (FDR < 20%). Putting these genes into enrichment analysis to identify hallmark pathways revealed 5 pathways unique to CIT myelin relative to UNMOD. Using an FDR threshold of 20% across these pathways, 691 unique genes were compiled and then clustered by average linkage and Pearson distance (Fig. 4B). This clustering revealed a group of 45 unique genes induced by citrullination relative to both vehicle and unmodified myelin. Of this cluster, 25 genes showed more than 1.2-fold increase and were significant by Tukey’s multiple comparison in a one-way ANOVA: ABCA1, B2M, BST2, C2, CCL5, CCRL2, CD274, CD38, CFB, GCH1, IGF2R, IL2RG, NAMPT, PIM1, PSMB10, PSMB8, PSME2, RRP12, RRP9, RSAD2, SOD2, TAPBP, TNF, TNFAIP3, UBE2L6. The 12 unique genes with the largest relative change in response to CIT myelin relative to UNMOD myelin are shown in Fig. 4B (*F*(2,6) = 47.61, *P* = 0.002 across groups by one-way ANOVA; CIT vs VEH: *P* = 0.002, CIT vs UNMOD: *P* = 0.0024 by Tukey’s multiple comparison test).

### Citrullinated myelin induces microglial TNFα and CCL5 responses

**Fig. 5:**
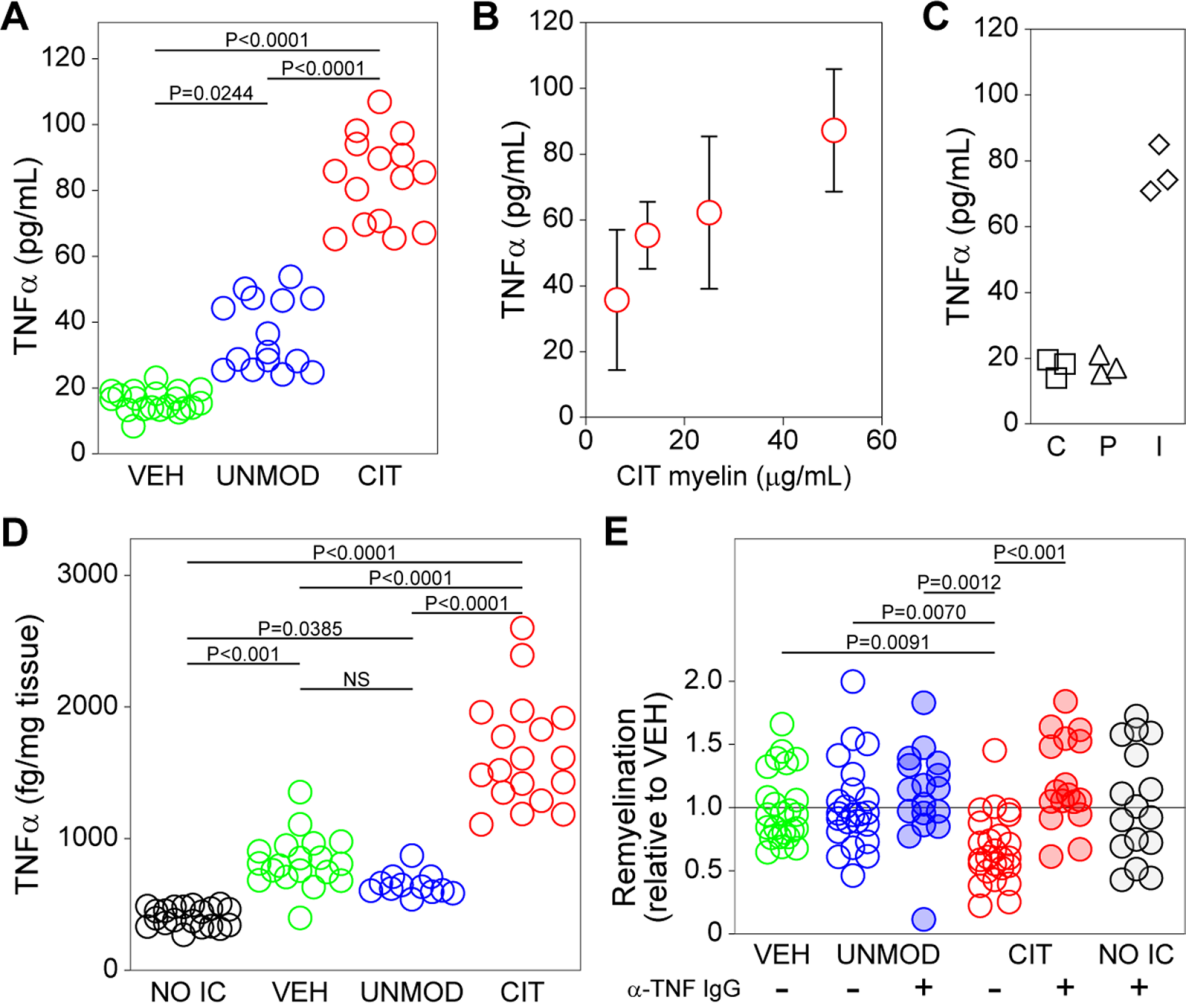
Neutralization of TNFα restores remyelination in mice injected with citrullinated myelin. **A** Primary microglia were incubated for 24 h with vehicle (VEH), unmodified myelin (UNMOD), or citrullinated myelin (CIT). TNFα levels were measured by cytometric bead array in supernatants. Each symbol represents one culture well replicate from several experiments. **B** Microglia were incubated with 50, 25, 12.5, or 6.25 μg/mL citrullinated myelin and TNFα levels were assessed after 24 h. **C** Microglia were exposed to pure citrulline (1.75 μg/mL), pure PAD2 enzyme (50 ng/mL), or IFNγ (200 ng/mL) and TNFα levels were assessed after 24 h. C = citrulline, P = PAD2, I = IFNγ **D** Mice were injected with VEH, UNMOD myelin, or CIT myelin and then a 4 mm diameter cylinder of cortical tissue around the injection site was extracted after 24 h and processed for measurement of TNFα levels. Each symbol represents one animal. **E** After 6 weeks on cuprizone chow, mice were switched to normal chow, injected intraperitoneally with TNFα neutralizing antibody (+ IgG) or isotype control (− IgG), and injected intracranially with UNMOD or CIT myelin. Remyelination was assessed by MBP-positive surface area in cortical sections 3 weeks later. A remyelination index was calculated by dividing the MBP surface area from each animal by the mean MBP surface area in all vehicle-injected mice. A control group receiving only anti-TNFα and no intracranial injection (NO IC) was included. Each symbol represents one animal. Graphs show mean ± 95% CI. Statistical comparisons not shown were insignificant

To determine whether the transcriptional changes were reflected in protein production, we stimulated primary microglia with vehicle or UNMOD or CIT myelin (50 µg/mL) and measured levels of CCL5 and TNFα in supernatants after 24 h. We found that both factors were increased by CIT myelin. CCL5 levels increased from 66 pg/mL in vehicle-treated cultures to 176 pg/mL in UNMOD and 340 pg/mL in CIT myelin stimulated microglia (*F*(2,31) = 10.82, *P* = 0.0003 across groups by one-way ANOVA; CIT vs VEH: *P* = 0.0002, UNMOD vs VEH: *P* = 0.1401, CIT vs UNMOD: *P* = 0.0243 by Tukey’s multiple comparison test). In parallel, we found that primary microglia incubated for 24 h with citrullinated myelin released 83 pg/mL TNFα, while cells exposed to unmodified myelin released 36 pg/mL. Cells exposed to vehicle released 6 pg/mL TNFα (*F*(2,47) = 217.6, *P* < 0.0001 across all groups by one-way ANOVA; CIT vs VEH: *P* < 0.0001, UNMOD vs VEH: *P* = 0.0244, CIT vs UNMOD: *P* < 0.0001 by Tukey’s multiple comparison test) (Fig. 5A). This effect was titratable, in that stimulation with less citrullinated myelin resulted in release of less TNFα (Fig. 5B). Moreover, the effect was not recapitulated by exposure of primary microglia to pure citrulline (1.75 μg/mL) or pure PAD2 enzyme (50 ng/mL); for reference, stimulation with interferon gamma (IFNγ) (200 ng/mL) for 24 h induced TNFα release that was comparable to that induced by CIT myelin (Fig. 5C).

The in vivo relevance of these findings was assessed by measuring TNFα and CCL5 transcripts in ribosome-associated RNA isolated from brain tissue collected at the site of myelin injection in CX3CR1^CreER^ x Rpl22 ribotag mice. At 24 h after injection of CIT or UNMOD myelin we observed no significant difference in either TNFα or CCL5 RNA levels between the groups using this approach (data not shown). Given the density of microglia in the tissue plug used for our analysis (~ 200 K microglia in a 40 mg plug) [37], we interpret this finding to suggest insufficient resolution of individual reactive microglia around the injection site. We therefore measured CCL5 and TNFα protein levels in similar tissue plugs. CCL5 levels were increased from 2 pg/mg in vehicle injected brain to 20 pg/mg in CIT myelin injected mice; delivery of UNMOD myelin had no effect over vehicle (*F*(2,20) = 9.259, *P* = 0.0014 by one-way ANOVA; CIT vs VEH: *P* = 0.0034, UNMOD vs VEH: *P* = 0.9741, CIT vs UNMOD: *P* = 0.0057 by Tukey’s multiple comparison test). TNFα levels increased from 0.8 pg/mg in vehicle-injected mice to 1.6 pg/mg in mice injected with CIT myelin (Fig. 5D). TNFα levels were not increased by UNMOD myelin (*F*(3,65) = 90.29, *P* < 0.0001 by one-way ANOVA; CIT vs VEH: *P* < 0.0001, UNMOD vs VEH: *P* = 0.1913, CIT vs UNMOD: *P* < 0.0001 by Tukey’s multiple comparison test). Notably, while the absolute size of the CCL5 response was larger than the TNFα response, we observed that the CCL5 induction was restricted to the ipsilateral cortex, while TNFα levels were also increased in the uninjected contralateral cortex in mice receiving CIT myelin. In cuprizone demyelinated mice, we also observed that the TNFα response to CIT myelin persisted in the ipsilateral cortex for at least 1 week after injection when assessed by immunostaining (Additional file 1: Fig. S3).

### Antibody-mediated neutralization of TNFα restores remyelination capacity in mice injected with citrullinated myelin

Finally, given the availability of clinically relevant drugs targeting TNFα, we treated cuprizone demyelinated mice with anti-TNFα neutralizing antibody (+ IgG) or an isotype control (− IgG) starting at the time myelin was injected into the cortex and animals were removed from cuprizone chow. Using a remyelination index calculated by normalizing the MBP surface area measurement in each animal after 3 weeks to the mean MBP surface area across all mice injected with vehicle (PBS), we found that anti-TNFα antibody had no impact on the remyelination capacity in mice receiving unmodified myelin (UNMOD + IgG vs UNMOD-IgG: *P* = 0.9688) but had a robust impact on remyelination in mice receiving citrullinated myelin (CIT + IgG vs CIT-IgG: *P* < 0.0001) (*F*(5,116) = 6.312, *P* < 0.0001 by one-way ANOVA; individual comparisons by Tukey’s pairwise test) (Fig. 5E). Moreover, TNFα neutralization increased the remyelination index in mice injected with citrullinated myelin to the same level measured in mice receiving vehicle (CIT + IgG vs VEH: *P* = 0.4229) and mice receiving unmodified myelin (CIT + IgG vs UNMOD + IgG: *P* = 0.9461) (Fig. 5E). We conclude that citrullinated myelin drives TNFα production by cortical microglia that suppresses endogenous remyelination.

## Discussion

As the resident phagocytes of the CNS, microglia play a key role in shaping inflammatory responses and strongly influence the ability of the brain to repair following pathogenic insult. Previous studies addressing the role of microglia-specific inflammation during demyelination implicated these inflammatory responses as both beneficial, via enhanced clearance of myelin debris, and detrimental, via inhibition of endogenous repair processes. To reconcile the differences in these conclusions, we hypothesized that post-translational biochemical modifications alter characteristics of myelin debris that dictate the nature of the inflammatory response induced in microglia. Our findings indicate that increased citrullination of major myelin proteins, as observed in patients with MS, drives a unique microglial inflammatory phenotype that negatively impacts the ability of the brain to remyelinate.

To determine the impact of myelin debris generated during active demyelination on endogenous repair processes, we isolated a myelin-enriched fraction from the homogenized cortex of adult mice using gradient centrifugation. This disrupted myelin fraction was then either used in an unmodified state or was exposed to the PAD2 enzyme in the presence of high calcium to citrullinate available proteins. To establish a model of endogenous myelin repair, we fed mice cuprizone-containing chow for 6 weeks to fully demyelinate axons that project through the corpus callosum and into the cortex and then returned the animals to normal chow for 3 weeks to allow remyelination to proceed. Within this context we injected unmodified or citrullinated myelin debris into the primary motor cortex just above the cingulum bundle that runs on top of the body of the corpus callosum. Myelin was delivered immediately after cessation of cuprizone chow and the impact of this material on spontaneous remyelination of the axons that fan out from the cingulum bundle was assessed after 3 weeks by immunostaining for the mature myelin factor MBP. While injection of unmodified myelin had no apparent effect on the extent of spontaneous remyelination at time of assessment, delivery of citrullinated myelin significantly inhibited this repair. Indeed, the amount of myelination measured at 3 weeks post-injection (3 weeks after cessation of cuprizone) in mice receiving citrullinated myelin was statistically indistinguishable from the amount of myelination present at the time cuprizone chow was stopped, while the amount of myelination in mice receiving unmodified myelin was statistically comparable to the myelination measured in mice that had never been treated with cuprizone.

Given the known role of microglia in the phagocytosis and degradation of damaged myelin [38], we assessed the microglial response to unmodified or citrullinated myelin injection. We found a large increase in Iba-1-positive microglia at 24 h around the site of injection in mice receiving both unmodified and citrullinated myelin and this activation was maintained through 72 h. Moreover, microglial activation was observed in the contralateral cortex in mice receiving either myelin, suggesting a non-local mechanism of activation. Because both myelin preparations activated microglia but only citrullinated myelin inhibited endogenous repair, we established an in vitro assay to test microglial responses to myelin. We found that both unmodified and citrullinated myelin were rapidly phagocytosed by purified primary microglia and both myelin preparations induced morphological changes consistent with activation. Moreover, we found that both myelin preparations induced changes in cellular markers consistent with activation, though citrullinated myelin drove a larger increase in MHCII and CD86 expression relative to unmodified myelin. RNAseq analysis of the microglial response to myelin revealed that both preparations induced a profound transcriptional change consistent with activation. However, citrullinated myelin induced a larger increase in a small number of genes, indicating a modification-dependent effect on the microglia. Of these genes, TNFα stood out as a possible effector mechanism to explain the impact of citrullinated myelin on spontaneous remyelination.

While exposure to either myelin preparation induced TNFα release from purified primary microglia, the amount of TNFα secreted by these cells in response to citrullinated myelin was at least twofold greater relative to unmodified myelin. Moreover, intracranial injection of unmodified myelin did not induce an increase in TNFα relative to vehicle injected mice, while delivery of citrullinated myelin drove a marked increase in TNFα levels around the injection site and in the contralateral cortex. Of note, the in vitro response to citrullinated myelin exhibited dose dependency and exposure of microglia to either citrulline or the PAD2 enzyme alone did not recapitulate the TNFα response induced by citrullinated myelin. Based on these findings, we asked whether neutralization of TNFα at the time of myelin injection would prevent the citrullinated myelin-induced inhibition of remyelination. We found that antibody-mediated neutralization of TNFα restored endogenous repair in mice receiving citrullinated myelin to the level observed in mice receiving unmodified myelin or vehicle. On this basis we conclude that citrullinated epitopes in myelin drive a unique microglial activation profile marked by TNFα production which consequently inhibits spontaneous remyelination.

Soluble TNFα signaling through TNFα receptor 1 (TNFR1) inhibits oligodendrocyte precursor cell maturation, induces oligodendrocyte death, and drives demyelination [39–43], while transmembrane TNFα signaling through TNFR2 drives pro-regenerative pathways, oligodendrocyte precursor cell differentiation [44], and an anti-inflammatory microglial phenotype [45]. This suggests that the anti-reparative effect of citrullinated myelin was likely mediated by soluble TNFα. From a clinical perspective, soluble TNFα levels are elevated in the cerebrospinal fluid of patients with progressive MS [46]. Unexpectedly, anti-TNFα therapy exacerbated disease in patients with MS [47, 48] and directly induced demyelination in some patients without MS [49]. While some of this effect may be due to changes in T regulatory cell-mediated immune suppression [50], it is likely that the primary driver of such iatrogenic effects was the indiscriminate neutralization of both soluble and transmembrane TNFα [51].

In support of this notion, specific inhibition of soluble TNFα promoted remyelination and ameliorated disease severity in several mouse models of demyelination [44, 52–54], while genetic deletion of both soluble and transmembrane TNFα exacerbated inflammation [55]. Genetic deletion of TNFR1 reduced disease severity and demyelination in the MOG experimental autoimmune encephalomyelitis model, while genetic deletion of TNFR2 profoundly increased demyelination in this model [55]. Within the context of our findings, antibody-mediated neutralization of TNFα had no detrimental effects on spontaneous remyelination in the cuprizone model but had a robustly positive effect in preventing the repair-suppressing effects of citrullinated myelin. If microglial activation and TNFα production in response to citrullinated myelin occurs in patients with MS, our findings suggest that pharmacological inhibition of soluble TNFα may enhance endogenous repair.

The extent and pathophysiological role of myelin citrullination in MS is poorly understood. Evidence suggests that about 20% of the 18.5 kDa species of MBP isolated from the healthy brain exhibits deimination at 6 of 19 possible arginines [56]. In myelin isolated from patients with MS, the citrullinated MBP fraction increases to 45% and in patients with the fulminant Marburg variant of MS, this increases to 80–90% [18, 19], with 18 of 19 arginines converted to citrulline. While such extensively modified myelin confers a host of pathophysiological outcomes, ranging from loss of myelin compression to increased proteolytic susceptibility, it is also possible that the citrullinated myelin elicits a feedback loop in which activated microglia produce inflammatory factors that drive myelin damage and increased extracellular calcium, leading to more myelin citrullination and more microglial activation [23]. In this context, it is notable that TNFα is highly upregulated within microglia containing myelin degradation products at the center of highly active lesions in patients with acute, fatal MS [57].

Both the Marburg variant and the Balo’s concentric sclerosis phenotype of MS are marked by a fulminant, aggressive, and frequently rapidly fatal disease course. While rare, these variants are poorly served by current therapeutic strategies. It is notable that lesions in patients with the Balo’s variant exhibit concentrically expanding rings of activated, myelin-laden microglia in proximity to dead and dying oligodendrocytes [58] and Marburg lesions are marked by myelin-laden CD68^+^ microglia/macrophages, myelin destruction, and intense inflammatory infiltrate [59, 60]. As discussed above, the Marburg variant is associated with extensive MBP citrullination; at present, no published evidence exists for an enrichment of this posttranslational modification in the Balo’s variant. However, evidence that hypercitrullination may proceed myelin damage in patients with early onset MS [61], coupled with the observation of elevated citrulline moieties in inflammatory demyelinating disorders such as acute disseminated encephalomyelitis [62] suggests that myelin citrullination may be associated with early active lesion development and/or aggressive therapy-resistant demyelination. In the context of our findings regarding the impact of citrullinated myelin on microglial activation, this suggests that anti-TNFα therapies should be reconsidered in specific MS patients with aggressive disease, perhaps as a “rescue” intervention to interfere with an acute microglial-driven inflammatory process. We hypothesize that transient neutralization of soluble TNFα will prevent expansion of demyelination in patients with the Balo’s phenotype and will reduce lesion burden in patients with the Marburg’s variant. Moreover, given that up to half of MBP molecules are citrullinated in patients with less aggressive forms of MS and current evidence suggests that other myelin proteins are also targets of deimination [63], our findings may have broad significance for treating a spectrum of MS patients. Likewise, the role for microglial activation and consequent inflammation-mediated disruption of axonal function and/or homeostasis [64] suggests that therapies which reduce TNFα may slow or inhibit disease progression in MS patients. While the field has struggled with previous TNFα targeting therapies in MS [48, 65, 66], a very cogent argument can be made (and indeed has been made [67]) that our deeper understanding of the complexities inherent to TNFα signaling calls for renewed efforts to therapeutically target soluble TNFα in patients with MS. An intriguing candidate for specifically inhibiting soluble TNFα without impairing protective juxtacrine TNFα signaling is the brain-permeant biologic XPro1595 [68], currently in phase 1b testing in patients with Alzheimer’s disease (NCT03943264). XPro1595 has already proven beneficial in MS models with increased preservation of myelin and axons [52] and improved phagocytosis of damaged myelin in lesions [68].

Targeting pathways upstream of TNFα may be a more viable therapeutic option for the wider MS population. PAD inhibitors that prevent citrullination may help stop the self-perpetuating cycle of inflammation and myelin damage. Several studies have already shown that PAD inhibitors attenuate disease in preclinical mouse models of MS [69–71] and in murine models of arthritis [72–74]. In fact, in a TNFα-induced model of arthritis, genetic deletion of PAD2 resulted in amelioration of joint inflammation [75], demonstrating the connection between PAD2 activity and TNFα-mediated destruction in another disease context. In a new model of demyelination termed cuprizone autoimmune encephalitis, Caprariello et al. demonstrated that PAD inhibition was capable of preventing the development of severe inflammatory cortical lesions [69]. Similarly, others have shown that PAD inhibition preserves myelin integrity in both cuprizone and experimental autoimmune encephalomyelitis [70, 71]. These studies establish that there is a strong connection between PAD expression and inflammation. Further studies will be necessary to determine whether such inhibitors will have value beyond preclinical models. In addition, studies to understand the signaling cascades that lead to TNFα release following microglial exposure to citrullinated myelin will likely reveal new, promising therapeutic targets for patients with MS.

## Conclusions

Citrullinated myelin drives a unique microglial phenotype that is marked by increased expression and release of TNFα. Intracranial injection of citrullinated myelin inhibits endogenous repair mechanisms involved in remyelination following cuprizone-induced demyelination. Antibody-mediated neutralization of TNFα prevented the inhibitory effect of citrullinated myelin and returned spontaneous remyelination to normal levels. Given the increase in myelin citrullination observed in tissues from patients with MS, our findings suggest that strategies aimed at reducing the microglial response to such modified myelin may confer therapeutic benefit, particularly in cases of fulminant, aggressive disease.

## Supplementary Information

**Additional file 1: Figure S1. **Citrullinated myelin-fed microglia do not influence OPC proliferation. **A** Primary microglia were treated with myelin products for 24 h, then co-cultured with GFP-labeled OPCs following a complete media change (green). **B** Twenty-four hours after co-incubation, OPC proliferation was assessed using the Click-iT Edu Proliferation Assay Kit. **Figure S2.** Injection of citrullinated myelin drives microglial activation. 72 h after injection of myelin, Iba-1+ reactive cells were colocalized with the microglia-specific marker TMEM119, as assessed by immunostaining. **Figure S3.** Relative to vehicle- or UNMOD myelin-injected mice, TNFα levels remain elevated in the cortex of cuprizone-demyelinated mice 1 week after injection of CIT myelin.

## Data Availability

The data sets used and/or analyzed during the current study are available from the corresponding author on reasonable request.

## Abbreviations

BSA: Bovine serum albumin
CBA: Cytometric bead array
CIT: Citrullinated myelin
CNS: Central nervous system
DMEM: Dulbecco’s modified Eagle medium
DTT: Dithiothreitol
EDTA: Ethylenediaminetetraacetic acid
FBS: Fetal bovine serum
FDR: False discovery rate
IFNƔ: Interferon gamma
LPS: Lipopolysaccharide
MBP: Myelin basic protein
MS: Multiple sclerosis
OPC: Oligodendrocyte precursor cell
PAD2: Peptidyl arginine deiminase
PBS: Phosphate-buffered saline
RPKM: Reads per kilobase per million mapped reads
TNFR1: Tumor necrosis factor alpha receptor 1
TNFα: Tumor necrosis factor alpha
UNMOD: Unmodified myelin
VEH: Vehicle
