# Supplementary material for: Citrullinated myelin induces microglial TNFα and inhibits endogenous repair in the cuprizone model of demyelination
Source: J Neuroinflammation. 2021 Dec 27;18:305. doi: 10.1186/s12974-021-02360-3 (PMC8711191; doi:10.1186/s12974-021-02360-3)
Supplement: Supplementary file 1 — Additional file 1: Figure S1. Citrullinated myelin-fed microglia do not influence OPC proliferation. A Primary microglia were treated with myelin products for 24 h, then co-cultured with GFP-labeled OPCs following a complete media change (green). B Twenty-four hours after co-incubation, OPC proliferation was assessed using the Click-iT Edu Proliferation Assay Kit. Figure S2. Injection of citrullinated myelin drives microglial activation. 72 h after injection of myelin, Iba-1+ reactive cells were colocalized with the microglia-specific marker TMEM119, as assessed by immunostaining. Figure S3. Relative to vehicle- or UNMOD myelin-injected mice, TNFα levels remain elevated in the cortex of cuprizone-demyelinated mice 1 week after injection of CIT myelin. [file 12974_2021_2360_MOESM1_ESM.docx]

**Additional file 1: Figure Legends:**

**Additional file 1:** Figure S1: Citrullinated myelin-fed microglia do not influence OPC proliferation. (A) Primary microglia were treated with myelin products for 24 hr, then co-cultured with GFP-labeled OPCs following a complete media change (green). (B) Twenty-four hours after co-incubation, OPC proliferation was assessed using the Click-iT Edu Proliferation Assay Kit.

**Additional file 1:** Figure S2: Injection of citrullinated myelin drives microglial activation. 72 hr after injection of myelin, Iba-1+ reactive cells were colocalized with the microglia-specific marker TMEM119, as assessed by immunostaining.

**Additional file 1:** Figure S3: Relative to vehicle- or UNMOD myelin-injected mice, TNFα levels remain elevated in the cortex of cuprizone-demyelinated mice 1 week after injection of CIT myelin.
